# Multi-Scale Landscape Influences on Genetic Diversity and Adaptive Traits in a Neotropical Savanna Tree

**DOI:** 10.3389/fgene.2020.00259

**Published:** 2020-03-25

**Authors:** Rosane Garcia Collevatti, Juliana Silveira dos Santos, Fernanda Fraga Rosa, Tatiana S. Amaral, Lazaro José Chaves, Milton Cezar Ribeiro

**Affiliations:** ^1^Laboratório de Genética & Biodiversidade, ICB, Universidade Federal de Goiás, Goiânia, Brazil; ^2^Laboratório de Ecologia Espacial e Conservação (LEEC), Departamento de Biodiversidade, Universidade Estadual Paulista Júlio de Mesquita Filho, Rio Claro, Brazil; ^3^Escola de Agronomia, Universidade Federal de Goiás, Goiânia, Brazil

**Keywords:** agroecosystem, Bignoniaceae, Cerrado, fragmentation, genetic diversity, landscape genetics, quantitative genetics, *Tabebuia aurea*

## Abstract

Changes in landscape structure can affect essential population ecological features, such as dispersal and recruitment, and thus genetic processes. Here, we analyze the effects of landscape metrics on adaptive quantitative traits variation, evolutionary potential, and on neutral genetic diversity in populations of the Neotropical savanna tree *Tabebuia aurea*. Using a multi-scale approach, we sampled five landscapes with two sites of savanna in each. To obtain neutral genetic variation, we genotyped 60 adult individuals from each site using 10 microsatellite loci. We measured seed size and mass. Seeds were grown in nursery in completely randomized experimental design and 17 traits were measured in seedlings to obtain the average, additive genetic variance (*V*_*a*_) and coefficient of variation (*CV*_*a*_%), which measures evolvability, for each trait. We found that habitat loss increased genetic diversity (*He*) and allelic richness (*AR*), and decreased genetic differentiation among populations (*F*_*ST*_), most likely due to longer dispersal distance of pollen in landscapes with lower density of flowering individuals. Habitat amount positively influenced seed size. Seeds of *T. aurea* are wind-dispersed and larger seeds may be dispersed to short distance, increasing genetic differentiation and decreasing genetic diversity and allelic richness. Evolvability (*CV*_*a*_%) in root length decreased with habitat amount. Savanna trees have higher root than shoot growth rate in the initial stages, allowing seedlings to obtain water from water tables. Landscapes with lower habitat amount may be more stressful for plant species, due to the lower plant density, edge effects and the negative impacts of agroecosystems. In these landscapes, larger roots may provide higher ability to obtain water, increasing survival and avoiding dying back because of fire. Despite the very recent agriculture expansion in Central Brazil, landscape changes are affecting neutral and adaptive variation in *T. aurea*. Several populations have low additive genetic variation for some traits and thus, may have limited evolvability, which may jeopardize species long-term persistence. The effect of habitat loss on highly variable neutral loci may only be detected after a certain threshold of population size is attained, that could become dangerously small masking important losses of heterozygosity endangering species conservation.

## Introduction

Agricultural landscapes are now occupying most of Neotropical savannas. The Brazilian Cerrado biome is the largest Neotropical savanna and one of the world’s biodiversity hotspots because of its high level of endemism and threatening (Myers et al., 2000). Over the last 50 years, more than 50% of its vegetation cover has been cleared or transformed into agriculture, pasture or urban area (Sano et al., 2010). Landscapes in the Cerrado biome are now comprised by islands of savanna surrounded by ocean of crops and pastures (see Latrubesse et al., 2019), thus jeopardizing species long-term conservation and ecosystem services maintenance.

Changes in landscape structure, such as habitat amount, edge effect and heterogeneity, can affect essential ecological processes such as dispersal (Corlett, 2017; Bovo et al., 2018), seed predation (Mendes et al., 2016), and pollination (Hadley and Betts, 2011; Duarte et al., 2018), and consequently compromise population persistence (Santos et al., 2016; Regolin et al., 2017). Landscape changes may also affect population connectivity and genetic diversity in different taxa (e.g., Dixo et al., 2009; Carvalho et al., 2015; Jackson and Fahrig, 2016; Moraes et al., 2018; González-Fernández et al., 2019). This occurs mainly due to reduction in population effective size and isolation (Gilpin and Soulé, 1986; Frankham et al., 1999). In this way, understanding how the still-ongoing landscape change affects savanna species may help designing sound conservation and management strategies.

In agricultural landscapes, anthropogenic matrix characteristics may be as important as habitat size and amount due to the potential restriction the agroecosystems may impose to species movement, dispersal and gene flow (Holderegger et al., 2010; Driscoll et al., 2013; Silva et al., 2015; Barros et al., 2019). Matrix type may affect functional connectivity (i.e., the degree to which the matrix facilitates or impedes species movement among habitat patches) because it may be filters or physical barriers to species’ movement (Ricketts, 2001; Shamoon et al., 2018). In plants, landscape structure may affect connectivity due to the influence on pollen dispersal, seed dispersal distance, germination and establishment (Damschen et al., 2014; Trakhtenbrot et al., 2014; Auffret et al., 2017). For instance, population size and productivity may decrease seed germinability, thus decreasing final seed dispersal distance due to habitat quality (Soons and Heil, 2002; Soons et al., 2004, 2005). Thus, although wind-dispersed species may be favored to some degree by habitat fragmentation – due to opened matrix increasing long-distance dispersal (Tackenberg et al., 2003) – changes in landscape composition and configuration may disrupt recruitment (Soons and Heil, 2002).

Most studies addressing the impacts of landscape composition and configuration in plant genetic diversity focus on genetic variation at neutral loci (e.g., Schmidt et al., 2009; Carvalho et al., 2015). Adaptive genetic variation can determine population persistence in the face of environmental changes (Levins, 1968; Meyers and Bull, 2002; Holderegger et al., 2006), such as the rapid landscape changes in the Anthropocene. Genetic variability at adaptive loci depends on the strength of selection and neutral molecular markers might be poor indicators of selection pressures at such loci (McKay and Latta, 2002). The loss of genetic variability at adaptive loci may lead to the loss of individual fitness and thus in population evolutionary potential (Lande, 1988; Reed and Frankham, 2002; Bijlsma and Loeschcke, 2012). Measurement of adaptive genetic variation often requires controlled experiments due to quantitative variation, that are not attainable for many species and thus is often overlooked in plant landscape genetics studies, hindering our understanding of the effects of land use in functional traits and adaption (Holderegger et al., 2006, 2010).

Here, we analyze the effects of landscape composition and configuration in variation at adaptive quantitative traits and at neutral genetic variability in populations of the Neotropical savanna tree *Tabebuia aurea* (Bignoniaceae); for this we used a multi scale approach. This species is broadly distributed across seasonal and wet savannas of Brazil, Bolivia and Paraguay, but locally it is distributed in well-delimited patches, usually with high density. It is hermaphroditic with mixed-mating reproductive system (Braga and Collevatti, 2011), pollinated by large-sized bees, such as carpenter bees and bumblebees, and seeds are wind-dispersed. Wind may promote long-distance seed dispersal in savanna trees with winged seeds, because long-distance seed dispersal by wind may be associated with plant height and seed morphology (Tackenberg et al., 2003). Because landscape structure may affect dispersal distance, seed germination and establishment we hypothesize that the relationships between our response variables (genetic variability, inbreeding and genetic differentiation) versus explanatory variables (habitat amount, compositional heterogeneity, matrix quality and connectivity) will have a vertex, i.e., a turning point. Away from the turning point, in landscapes with very high or very low habitat amount, compositional heterogeneity, matrix quality and connectivity we expect that genetic variability at neutral and adaptive loci will be lower and genetic differentiation and inbreeding will be higher. Conversely, in landscapes with average habitat amount, compositional heterogeneity, matrix quality and less isolated patches genetic variability at neutral and adaptive loci will be higher and genetic differentiation and inbreeding will be lower. We also hypothesize that population evolvability will be reduced due to habitat and connectivity losses following the trend expected for genetic variability. Because genetic variability is affected by effective populations size and changes in adaptive traits may be a balance between drift and selection (Wright, 1931; Kimura, 1983), we also analyze the effect of effective population size in adaptive and neutral genetic variation.

## Materials and Methods

### Study Sites and Landscape Sampling Design

**FIGURE 1:**
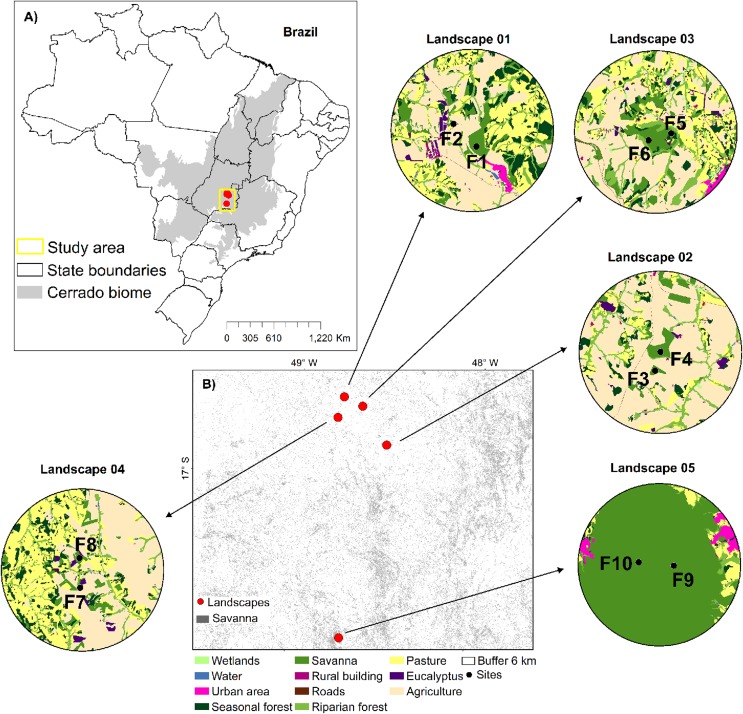
Geographic distribution of the five landscapes and the 10 sampling sites of *Tabebuia aurea* in the Brazilian Cerrado. **(A)** The distribution of the Cerrado biome in Brazil and the landscapes sampled. **(B)** The landscapes represented by a buffer of 6 km and the sampling sites (black dots). Land use categories are in legends. For details of sampling sites see Appendix Table S1.

The study was carried out in five landscapes of savanna in the Cerrado biome in Goiás state, Central-West Brazil (Figure 1 and Appendix Table S1), one of the most important Brazilian agribusiness regions. Each landscape was defined by a buffer of 6 km radius, where two sites of savanna with populations of *T. aurea* were sampled (total of 10 sites). The sites were either structurally isolated in different savanna fragments (P1, P2, and P4, Figure 1 and Appendix Table S1) for many landscapes, or in the same large savanna protected area (P3 and P5, Figure 1 and Appendix Table S1). In any case, we selected sampling sites spaced apart by at least 1.4 km (Appendix Table S1).

We mapped land cover in these five landscapes using high-resolution images available at the map database of the Geographic Information System in ArcGis v.9.3 environment (Esri^®^). Mapping was performed using visual digitalization and manual classification at a scale of 1:5,000 m, and we made exhaustive field inspection to assure high map accuracy. Our final map comprised 11 different land cover classes: (i) water courses; (ii) savanna; (iii) riparian forest; (iv) seasonal forest; (v) wetland; (vi) pasture; (vii) agriculture (corn or soybean); (viii) rural building; (ix) urban area; (x) road and train rail, and (xi) *Eucalyptus* spp. plantation (Figure 1).

### Genetic Data

#### Genetic Analysis for Neutral Loci

To obtain genetic parameters for neutral loci, we sampled expanded leaves of ∼60 individuals of *T. aurea* in each site (Appendix Table S2). In sites with less than 60 we sampled all individuals. Total genomic DNA extraction followed CTAB procedure and all individuals were genotyped using 10 microsatellite loci (Braga et al., 2007) following PCR protocol described in Collevatti et al. (2014). DNA fragments were sized in GS 3500 Genetic Analyzer (Applied Biosystems, CA) using GeneScan ROX 500 size standard (Applied Biosystems, CA), and were scored using GeneMaper v5.0 software (Applied Biosystems, CA). Genotyping errors (allele dropout and null allele) were analyzed using Micro-Checker 2.2.3 software (van Oosterhout et al., 2004).

For each site (10), we estimated genetic diversity (expected heterozygosity under Hardy-Weinberg equilibrium, *He*; Nei, 1978), allelic richness based on rarefaction analysis (*AR*; Mousadik and Petit, 1996) and inbreeding coefficient (*f;* Wright, 1951). All analyses and randomization-based tests for deviation from Hardy-Weinberg equilibrium were performed with the software FSTAT 2.9.3.2 (Goudet, 2002). We estimated genetic differentiation among all pairs of sites nested within landscapes (5) using Wright’s *F*_*ST*_, and fixation index (inbreeding) *F*_*IS*_ obtained from an analysis of variance of allele frequencies (Weir and Cockerham, 1984). We also estimated *G_*ST*_’* (Hedrick, 2005) that is based on *F_*ST*_*, but takes into account observed diversity within population and number of subpopulations, and Jost’ *D* (Jost, 2008) that is based on effective number of alleles instead of expected heterozygosity. To estimate the contribution of stepwise mutation model to genetic differentiation, we estimated Slatkin’s *R*_*ST*_ (Slatkin, 1995) and tested the hypothesis that *F_*ST*_* = *R*_*ST*_ using the software Spagedi (Hardy and Vekemans, 2002).

We used microsatellite markers to estimate effective population size (*N*_*e*_). We used the molecular co-ancestry method (Nomura, 2008) implemented in NeEstimator V2.1 (Do et al., 2014) and tested the hypothesis that genetic variability and differentiation depend on *N*_*e*_ (see below).

#### Adaptive Quantitative Trait Variation

We obtained quantitative genetic variation for traits related to the fitness of seeds and seedlings, i.e., adaptions related to the initial establishment of seedlings. We focused on seeds and seedlings because these stages are related to plant dispersal and establishment, and seedling is the stage at which plants are most vulnerable to the effects of environment variables (Tomlinson et al., 2012).

We measured 20 quantitative traits related to seed size (longitudinal and transversal diameter) and mass and to seedling growth (height and diameter growth), size (height, leaf size and number, shoot and root length) and mass (root and aboveground). Seed size and mass are strongly related to seedling successful establishment and also with other adaptive traits such as plant height and specific leaf area (Westoby et al., 1996). Height and leaf traits are frequently used to assess the variation in plant ecological strategies because they represent plant response to stress and resource use (Westoby, 1998; Reich, 2000; Westoby et al., 2002). Plant height is related to competitive capacity or disturbance avoidance (Westoby, 1998), which is important in savannas where fire events are frequent and taller plants may avoid top kill due to the death of canopy meristem (Hoffmann et al., 2012). Leaf area represents a functional strategy related to resource retention and photosynthesis rate (Reich et al., 1992; Westoby, 1998; Ackerly et al., 2002; Perez-Harguindeguy et al., 2013) and dry mass is related to the regulation of water loss through evapotranspiration (Poorter et al., 2009), increasing water use efficiency in low moisture soils (Ackerly, 2004; Poorter et al., 2009).

To obtain quantitative genetic variation, we sampled seeds from randomly chosen trees in each site, obtaining a total of 1070 seeds (Appendix Table S2). The number of trees sampled differed due to the number of adults siring fruits. Seeds were measured and weighted to obtain seed traits, SLD (seed longitudinal diameter, mm), STD (seed transversal diameter, mm) and SM (seed mass, mg) (Appendix Table S3). Then, seeds were grown in nursery in a completely randomized experimental design. The number of seedlings analyzed per tree per site differed due to variation in germination (Appendix Table S2). We also obtained the number of days to shoot (time to seed germination, TG) and the proportion of seeds that germinated (PG).

Plant aboveground height and stem diameter were measured 76, 116, 133, and 145 days after seed germination, and growth rate was obtained from the regression coefficient (β) of height (HGR, cm/day) and diameter (DGR, mm/day). We also registered the initial and final seedling height (IH and FH, cm) and diameter (ID and FD, mm). We count the total number of leaves (NL), and leaf length (LL, mm) and width (LW, mm) were obtained from the mean value among three leaves per seedling, measured up to 145 days after germination (Appendix Table S3). After 145 days, seedlings were taken from the nursery pots to measure (Appendix Table S3) aboveground shoot length (ASL, cm), root length (RL, cm). We also obtained aboveground green mass (AGM, g) and dry mass (ADM, g), and root green (RGM, g) and dry masses (RDM, g).

For statistical analyses, we first performed Pearson correlation analyses among quantitative variables for sites and landscapes (Appendix Tables S4, S5), to minimize the correlation in our set of quantitative traits. Therefore, for the subsequent analyses, we excluded the quantitative traits with correlation coefficient *r* > 0.5. We kept for seed traits SLD, STD and SM, and for seedlings, NL, LW, RL, RDM, and ASL.

We estimated the variance (*V*_*a*_) and the additive genetic coefficient of variation (*CV*_*a*_%), for each selected trait in each site, as a surrogate of population evolutionary potential or evolvability (Houle, 1992; Hansen et al., 2011). We also obtained narrow-sense heritability (*h*^2^) of each trait in each site. These parameters were estimated using restricted maximum likelihood (REML) analysis implemented in the software SELEGEN–REML/BLUP (Resende, 2016). We used model 82, implemented for open-pollinated sib families and mixed mating system. In this model, additive genetic variance is corrected by the coefficient $c=2\,\theta =\frac{{\left(1+s\right)}^{2}}{2\,\left(2-s\right)}$. For this, we estimated θ, the coancestry coefficient within family (Vencovsky et al., 2001; Tambarussi et al., 2018), $\theta =\frac{{\left(1+3\,f\right)}^{2}}{8\,\left(1+f\right)}$, and the selfing rate $s=\frac{2\,f}{1+f}$ (Vencovsky and Crossa, 2003), *f* is the inbreeding coefficient within population. Inbreeding was obtained from neutral loci (see above). To estimate additive genetic variance and the other parameters (*CV_*a*_%* and *h*^2^) we used $\sigma_{A}^{2}=\frac{1}{2\,\theta }\,\sigma_{p}^{2}$, where $\sigma_{p}^{2}$ is the genetic variance among families. Because seed traits were measured in seeds sampled in the field, we cannot estimate additive genetic variation. Thus, these parameters were estimated only for seedling traits obtained from controlled experiment in nursery.

To obtain the additive quantitative differentiation between pair of sites nested within landscapes, we estimated *Q*_*ST*_ (Spitze, 1993; Prout and Barker, 1993) and the analogous *P*_*ST*_ (Leinonen et al., 2006) for each selected trait. *Q*_*ST*_ estimates quantitative genetic differentiation based on population additive genetic variation (Spitze, 1993), and was estimated for seedling traits measured in nursery under experimental conditions. *P*_*ST*_ is analogous to *Q*_*ST*_ (Leinonen et al., 2006), but estimates phenotypic differentiation when genetic and environmental effects cannot be detached, due to uncontrolled environmental conditions. We estimated *P*_*ST*_ for seed traits because they were sampled in the field, under uncontrolled environmental conditions. *Q*_*ST*_ and *P*_*ST*_ parameters were estimated using model 5 implemented in SELEGEN–REML/BLUP software (Resende, 2016), also correcting the additive genetic variance for mixed mating system (see above).

### Landscape Metrics

**FIGURE 2:**
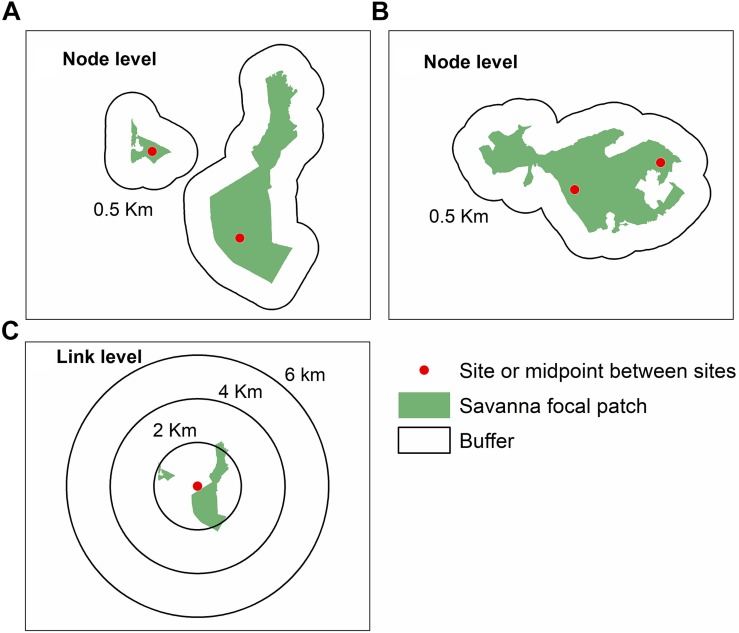
Sampling design to quantify landscape metrics at node and link levels for *Tabebuia aurea* in the Brazilian Cerrado. **(A)** Sampling design at node level in landscapes with sites in different patches of savanna. **(B)** Sampling design at node level in landscapes within the same patch of savanna, which corresponded to protected areas. **(C)** Sampling design at link level showing the buffers with different radii around the midpoint between the two sampling sites. For details of sampling sites see Figure 1, and Appendix Table S1.

We quantified landscape variables at node and link levels (Figure 2), following Wagner and Fortin (2013) framework. To calculate landscape metrics at the node level, we generated buffers of 0.5 km around each focal site (Figure 2A). We chose this minimum distance to avoid overlap between buffers. For the sites in protected areas (landscapes P3 and P5, Figure 1), we considered the same values for landscape metrics (Figure 2B).

To perform the analysis at the link level we identified the midpoint between the sampling sites, and performed multi-scale analysis, generating different buffers of 2, 4, and 6 km radius around each midpoint (Figure 2C). We chose the minimum buffer size because of pollen dispersal distance (at least 2 km, Braga and Collevatti, 2011). Additionally, buffer of 6 km of radius corresponded to the maximum size to avoid overlap at the link level (Figure 1).

To characterize landscape structure we quantified different landscape metrics related to the composition and configuration of landscape elements. As savanna is considered an essential vegetation type to the establishment of *T. aurea*, we estimated the total habitat amount calculating the percentage of savanna at both node and link levels at different spatial scales (Appendix Tables S6, S7). To avoid correlation between habitat amount at different spatial scales at the link level, we subtracted the percentage of savanna in the additional scales and considered these new values as an additional metric. For instance, the metric “% savanna 4_2km” corresponded to the percentage of savanna in the scale of 4 km minus the percentage of savanna in the scale of 2 km (Appendix Table S7). To account for the effect of pollinators’ habitat, we summed up the amount of open vegetation where *T. aurea* can eventually establish, besides savanna (pasture, wetlands) and pollinators’ habitat (seasonal and riparian forests) to generate the variables: (i) % savanna + pasture + wetlands and (ii) savanna + pasture + seasonal forest + riparian forest + wetlands. We also avoided correlation subtracting the values of each metric in additional scales (Appendix Table S7).

The compositional heterogeneity was calculated using the Shannon index (SHDI) available in Fragstats (McGarigal et al., 2012), which included all land cover types within the buffers (Appendix Tables S6, S7). To estimate the functional landscape connectivity we selected all patches of savanna at different spatial scales and made individual buffers of 500 m of radius around each patch. We chose this radius due to foraging distance of potential pollinators of *T. aurea* such as *Bombus* spp. and *Centris* spp. (Braga and Collevatti, 2011). We considered as functional connectivity the sum of the area in hectares of all patches of savanna plus the area connected by the buffers (Appendix Tables S6, S7). The functional landscape connectivity was calculated using GRASS GIS 7.5 (GRASS Development Team, 2018).

To address matrix quality we used the Buffer Matrix Quality (BMQ) metric (Lion et al., 2014; see also Silva et al., 2015), *BMQ* = ∑*P*_*i*_*Q*_*i*_/∑*P*_*i*_, where *P*_*i*_ is the percentage of each land cover class in each landscape at different spatial scales, and *Q*_*i*_ is the quality score of each land cover class considering the requirements and resources that each land cover can provide to focal species. We scored each land cover based in the characteristics of *T. aurea’s* pollinators, since *T. aurea* is wind-dispersed and seedlings can eventually establish in wetlands and grasslands. For this, we considered our personal experience, the literature and the opinion of other experts in bees’ behavior. Our scores values ranged from the 0.2 to 1.0, where low values corresponded to areas more impermeable to species (Appendix Tables S6, S7).

For landscape variables at the link level, we first assessed the scale of effect calculating Pearson correlation between the same landscape metrics at different spatial scales. Because all correlations were high (i.e., *r* > 0.90), we kept only metrics for 2 km spatial scale. We then assessed collinearity between landscape metrics within 2 km spatial scale (Appendix Table S8). At the node level we calculated correlation coefficients between all landscape metrics at 0.5 km spatial scale (Appendix Table S9). We excluded from the subsequent analyses the landscape metrics with correlation coefficient *r* > 0.5. At node level, we kept habitat amount (%) and compositional heterogeneity (Appendix Table S9). At link level, we used habitat amount at 2 km spatial scale (Appendix Table S8).

### Data Analysis

We analyzed the effects of landscape metrics using Generalized Linear Model (GLM) for both neutral genetic parameters and adaptive quantitative traits (Table 1). Because genetic variability may be affected by effective population size (*N*_*e*_), we modeled genetic parameters using both landscape metrics and *N*_*e*_ (Table 1).

**TABLE 1:** Models performed at node and link levels for both neutral and adaptive quantitative traits measured in seeds and seedlings of *Tabebuia aurea* from 10 sites and 5 landscapes in the Brazilian Cerrado.

Analysis level	Response variable	Predictor variable
Node	**Neutral genetic variability** Genetic diversity (*He*) Allele richness (*AR*) Inbreeding coefficient (*f*)	**Landscape – 500 m** Habitat amount (%) Composition heterogeneity
		**Genetic** Effective population size (*Ne*)
	**Adaptive quantitative variation** SLD SDT SM NL LW RL SDM ASL	**Landscape – 500 m** Habitat amount (%) Compositional heterogeneity **Genetic** Effective population size (*N*_*e*_)
	**Evolvability** *CV*_*a*_%NL *CV*_*a*_%LW *CV*_*a*_%RL *CV*_*a*_%RDM *CV*_*a*_%ASL	**Landscape – 500 m** Habitat amount (%) Compositional heterogeneity **Genetic** Effective population size (*N*_*e*_)
Link	**Neutral genetic differentiation** *F*_*ST*_ *G*_*ST*_ Jost’D Inbreeding coefficient (*F*_*IS*_)	**Landscape – 2, 4 and 6 km** Habitat amount 2 km (%)
	**Adaptive quantitative variation** SLD STD SM NL LW RL SDM ASL	**Landscape – 2, 4 and 6 km** Habitat amount 2 km (%)
	**Adaptive quantitative differentiation** *P*_*ST*_ – SLD *P*_*ST*_ – STD *P*_*ST*_ – SM *Q*_*ST*_ – NL *Q*_*ST*_ – LW *Q*_*ST*_ – RL *Q*_*ST*_ – RDM *Q*_*ST*_ – ASL	**Landscape – 2, 4 and 6 km** Habitat amount 2 km (%)

*The values of genetic parameters are the average for each sampling site or landscape, or the additive genetic coefficient of variation (CV_a_%). SLD, seed longitudinal diameter (mm); STD, seed transversal diameter (mm); SM, seed mass (mg); NL, number of leaves; LW, leaf width (mm); RL, root length; RDM, root dry mass; ASL, aboveground shoot length (plant height).*

We evaluated the distribution of the residuals of all models and used the Gaussian distribution that fitted better to our data. To find the best predictive model at node level we considered both the significance and the Akaike Information Criteria (AIC). We estimated AIC corrected for small sample sizes (AICc) and the difference of each model and the best model (ΔAICci). We also estimated Akaike’s Weight of Evidence (wAICc) as the relative contribution of each model to explain the observed pattern, given a set of competing models (Burnhan and Anderson, 2002). Models with ΔAICc < 2 were considered as equally plausible to explain the observed pattern (Zuur et al., 2009).

For link level, we used the significance to select the best predictive model, because of the small sample size (five landscapes). Additionally, the sampling sites in P5 (F9 and F10, Figure 1 and Appendix Table S1) had outlier behavior in the analysis performed at the link level for neutral loci, hindering model fitting. To minimize this effect, we log transformed (log10) predictor variables. All statistical analyses were performed with R version 3.6.1. (R Core Team, 2019) using the following packages *bblme* (Bolkerand et al., 2017), *visreg* (Breheny and Burchett, 2017), and *ggplot2* (Wickham, 2016).

## Results

### Neutral Genetic Diversity and Differentiation

All loci had high genetic diversity and allelic richness (Appendix Table S10). Genetic diversity (*He*) was high in all populations ranging from 0.874 to 0.921 (Appendix Table S10). Allelic richness (*AR*) was also high, ranging from 14.2 to 18.5 (Appendix Table S10). Inbreeding coefficient (*f*) was significant for all populations, ranging from 0.121 to 0.231. *F*_*ST*_ across landscapes ranged from 0.008 to 0.240, *G*_*ST*_’ ranged from 0.115 to 0.304, and Jost’ *D* ranged from 0.104 to 0.277 (Appendix Table S11). Slatkin’s *R*_*ST*_ (*R_*ST*_* = 0.077, *SE* = 0.048, *p* < 0.001) was not significantly different from *F*_*ST*_ (*p* = 0.852). Inbreeding coefficients within landscapes (*F*_*IS*_) was also high (Appendix Table S11). All populations showed low effective population sizes (Appendix Table S10), ranging from 19.7 to 56.0.

### Adaptive Quantitative Trait Variation

Overall, populations had high additive genetic variance (V_*a*_) in quantitative traits, but some had low variance in several traits (e.g., P2F4, Appendix Table S12), leading to low values of additive genetic coefficient of variation and narrow-sense heritability (Appendix Table S12). For instance, *V*_*a*_ ranged from 0.0479 to 25.9293 for leaf width, and 0.0025 to 0.6116 for root dry mass (Appendix Table S12). Narrow-sense heritability for seedling traits showed a wide range among sites (Appendix Table S12), ranging from 0.0045 to 0.9419 for leaf width, 0.0024 to 0.6877 and for root dry mass, for example. Genetic differentiation in quantitative traits (*Q*_*ST*_) was low in most of the landscapes (Appendix Table S13), ranging from 0.005 (RDM) to 0.8140 (RL). Phenotypic differentiation in seed traits (*P*_*ST*_) was also low for most pairwise comparisons, ranging from 0.0003 to 0.0835 (Appendix Table S13).

### Landscape Effect on Neutral Genetic Diversity and Differentiation

**FIGURE 3:**
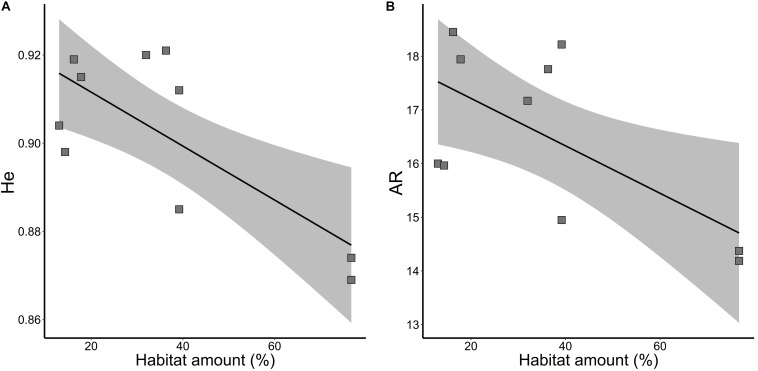
Relationships of neutral genetic variability and habitat amount in five landscapes and 10 sampling sites of *Tabebuia aurea* in the Brazilian Cerrado. **(A)** Genetic diversity (*He*). **(B)** Allelic richness (AR). Black line is the linear regression fit and shaded area is the 95% confidence interval. All fits were significant (*p* < 0.05).

Habitat amount was the model that best explained the variation in neutral genetic diversity (*He*, wAICc = 0.94; Table 2) and allelic richness (*AR*, wAICc = 0.87; Table 2). Genetic diversity (Figure 3A) and allelic richness (Figure 3B) decreased with habitat amount. None of the models tested was significant for inbreeding (*f*, all *p* > 0.10).

**TABLE 2:** Model selection of the competing hypotheses to explain the patterns of variation in neutral genetic variability and adaptive quantitative traits, in populations of *Tabebuia aurea* in landscapes of the Brazilian Cerrado.

Models	*He*				
	AICc	ΔAICc	df	wAICc	*p*
Habitat amount	−49.50	**0.00**	3.00	0.94	0.013**
Effective population size (*N*_*e*_)	−42.80	6.70	3.00	0.03	0.300
Compositional heterogeneity	−41.70	7.80	3.00	0.01	0.640

	***AR***				
	**AICc**	**ΔAICc**	**df**	**wAICc**	***p***

Habitat amount	41.60	**0.00**	3.00	0.87	0.043**
Effective population size (*N*_*e*_)	46.70	5.10	3.00	0.06	0.640
Compositional heterogeneity	46.80	5.20	3.00	0.06	0.710

	**SLD**				
	**AICc**	**ΔAICc**	**df**	**wAICc**	***p***

Habitat amount	53.1	**0.00**	3.00	0.65	0.099*
Effective population size (*N*_*e*_)	55.5	2.40	3.00	0.20	0.333
Compositional heterogeneity	56.0	2.90	3.00	0.15	0.474

	**NL**				
	**AICc**	**ΔAICc**	**df**	**wAICc**	***p***

Effective population size (*N*_*e*_)	17.1	**0.00**	3.00	0.74	0.077*
Habitat amount	20.5	3.40	3.00	0.14	0.443
Compositional heterogeneity	20.7	3.60	3.00	0.12	0.509

	***CV*_*a*_% – NL**				
	**AICc**	**ΔAICc**	**df**	**wAICc**	***p***

Habitat amount	76.8	**0.00**	3.00	0.70	0.084*
Effective population size (*N*_*e*_)	79.4	2.60	3.00	0.19	0.322
Compositional heterogeneity	80.3	3.50	3.00	0.12	0.569

	***CV*_*a*_% – RL**				
	**AICc**	**ΔAICc**	**df**	**wAICc**	***p***

Habitat amount	61.1	**0.00**	3.00	0.48	0.064*
Compositional heterogeneity	61.6	0.50	3.00	0.37	0.082*
Effective population size (*N*_*e*_)	63.4	2.3	3.00	0.15	0.198

	***CV*_*a*_% – RDM**				
	**AICc**	**ΔAICc**	**df**	**wAICc**	***p***

Compositional heterogeneity	86.1	**0.00**	3.00	0.83	0.062*
Habitat amount	90.6	4.50	3.00	0.08	0.781
Effective population size (*N*_*e*_)	90.7	4.60	3.00	0.08	0.893

He, genetic diversity; AR, allelic richness; f, inbreeding coefficient; SLD, seed longitudinal diameter; NL, number of leaves; CV_a_% -NL, additive genetic coefficient of variation of number of leaves, CV_a_% -RL of root length; CV_a_% -RDM, of root dry mass. ** significant (p < 0.05); * marginally significant (p < 0.10). AICc, AIC corrected for sample size and number of parameter in the model; wAICc, Akaike’s weight of evidence. Models with **Δ**AICc < 2.0 and significant are in bold.

**FIGURE 4:**
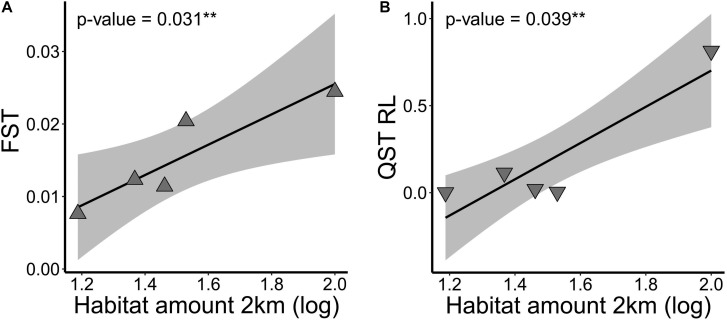
Relationship of **(A)** genetic differentiation at neutral loci (*F*_*ST*_) and at **(B)** adaptive quantitative trait (*Q*_*ST*_) root length (RL), and habitat amount at 2 km spatial scale in five landscapes of *Tabebuia aurea* in the Brazilian Cerrado. Black line is the linear regression fit and shaded area is the 95% confidence interval. Fits were significant (*p* < 0.05).

At link level, genetic differentiation based on *F*_*ST*_ was significantly related to habitat amount (β = 0.021; *p* = 0.031). Populations within landscapes with higher amount of habitat had higher genetic differentiation (Figure 4A). *G’_*ST*_* and Jost’s *D* were not explained by our model (*p* > 0.10). Inbreeding coefficient (*F*_*IS*_) was also not significantly related to habitat amount.

### Landscape Effect on Adaptive Quantitative Traits

**FIGURE 5:**
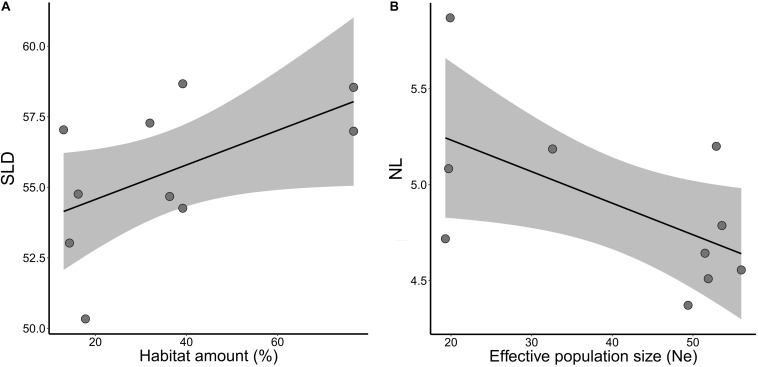
Relationships of adaptive quantitative traits and habitat amount and effective population size (*Ne*) in five landscapes of *Tabebuia aurea* in the Brazilian Cerrado. **(A)** Mean seed longitudinal diameter (SLD) and habitat amount. **(B)** Mean number of leaves (NL) and effective population size (*N*_*e*_). Black line is the linear regression fit and shaded area the 95% confidence interval. All fits were marginally significant (*p* < 0.10).

Habitat amount explained variation in seed size (SLD) between sites within landscapes, although marginally significant (wAICc = 0.65, *p* = 0.099; Table 2). Sites in landscapes with higher amount of habitat tended to have larger seeds (Figure 5A). Number of leaves (NL) was better explained by effective population size (*N_*e*_*, wAICc = 0.74) than by landscape structure, but was marginally significant (*p* = 0.077; Table 2). Number of leaves was negatively related to *N*_*e*_ (Figure 5B). Landscape structure and *N*_*e*_ did not explain variation in mean STD, SM, RL, RDM, and ASL between sites within landscapes (all *p* > 0.10).

**FIGURE 6:**
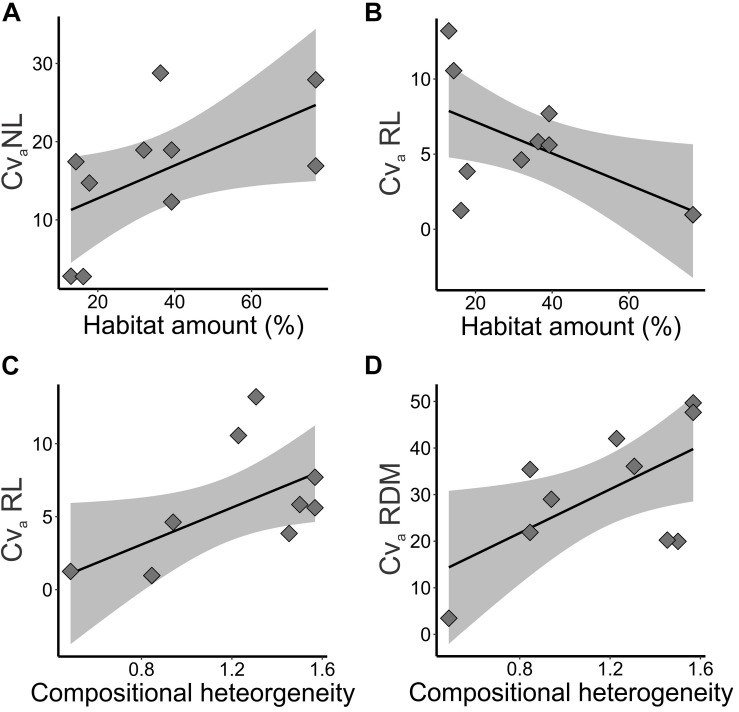
Relationships of additive genetic coefficient of variation (*CV*_*a*_%) of adaptive quantitative traits and landscape features in five landscapes and 10 sampling sites of *Tabebuia aurea* in the Brazilian Cerrado. **(A)** Additive genetic coefficient of variation (*CV*_*a*_%) in number of leaves (NL) and habitat amount. **(B)** Additive genetic coefficient of variation (*CV*_*a*_%) in root length (RL) and habitat amount. **(C)** Additive genetic coefficient of variation (*CV*_*a*_%) in root length (RL) and compositional heterogeneity. **(D)** Additive genetic coefficient of variation (*CV*_*a*_%) in dry root mass (RDM) and compositional heterogeneity. Black line is the linear regression fit and shaded area is the 95% confidence interval. Fits were significant (*p* < 0.05) or marginally significant (*p* < 0.10).

Landscape structure explained evolvability of seedling traits (Table 1), measured by the additive genetic coefficient of variation (*CV*_*a*_%). NL (wAICc = 0.70, *p* = 0.084) and RL (wAICc = 0.48, *p* = 0.064) coefficients of variation were better explained by habitat amount (Table 2). Coefficient of variation in NL increased with habitat amount (Figure 6D), while RL coefficient of variation decreased with habitat amount (Figure 6A). RL (wAICc = 0.37, *p* = 0.082) and RDM (wAICc = 0.83, *p* = 0.062) coefficients of variation were explained by compositional heterogeneity, although marginally significant (Table 2), and both tended to increase with compositional heterogeneity (Figures 6B,C).

At link level, genetic differentiation at root length (RL) based on *Q*_*ST*_ was significantly related to habitat amount (b = 1.041; *p* = 0.039). Populations within landscapes with higher amount of habitat had higher quantitative genetic differentiation in RL (Figure 4B). Our models did not explain (*p* > 0.10) differentiation at other seedling or seed adaptive traits.

## Discussion

### Pollen Dispersal May Explain the Relationships of Habitat Amount and Neutral Genetic Diversity and Differentiation

Our findings show that contemporary changes in landscape affect genetic diversity and differentiation at neutral microsatellite loci among populations of *T. aurea* from the Cerrado biome. However, unexpectedly, habitat loss increased genetic diversity and allelic richness and decreased genetic differentiation among populations. This result is most likely due to pollinator behavior and pollen dispersal patterns. *Tabebuia aurea* has a mixed mating system, with potential long-distance pollen dispersal of at least 2.6 km (Braga and Collevatti, 2011). Pollinators of this species, mainly bumblebees and carpenter bees, have long distance flight capacity, and may carry pollen over long distances (Goulson and Stout, 2001). However, in mass-flowering species, such as *T. aurea*, the residence time of pollinators may be greater decreasing pollen carry-over and thus effective population size (Frankie et al., 1976). Therefore, the mass-flowering pattern added by the clumped distribution of individuals within populations may affect pollinator foraging behavior, increasing pollinator residence time and leading to a high proportion of low-distance pollen dispersal (Braga and Collevatti, 2011). Habitat loss may decrease population size and density in *T. aurea*, leading to lower number of individuals flowering within populations, which may decrease the frequency of pollination between nearest neighbors and increase pollen dispersal distance (Kitamoto et al., 2006). In fact, populations in protected areas, such as the landscapes P3 and P5, with the highest habitat amount and structural connectivity, showed the lowest values of genetic diversity and allelic richness, but the highest genetic differentiation for the three parameters (*F_*ST*_, G’_*ST*_* or Jost’s *D*).

Our results contrast to other studies with *T. aurea* (Collevatti et al., 2014) and other savanna tree species (e.g., *Caryocar brasiliense*, Collevatti et al., 2001) that found no correlation between genetic diversity and habitat loss. This is most likely due to the spatial scale, since the former studies were performed in biome scale, where phylogeographic and biogeographic factors may be more important than local process of landscape changes.

Our findings also evinced high inbreeding within populations and high fixation index (*F*_*IS*_) within landscapes, but not related to landscape structure. In fact, *T. aurea* have high proportions of self-pollination and biparental inbreeding (Braga and Collevatti, 2011) and high inbreeding in several populations throughout its geographical distribution (Collevatti et al., 2014), which may explain the high values found in this work. Gene dispersal based on seedling fine scale genetic structure is low (nearly 300 m, Braga and Collevatti, 2011), which may also explain the lower differentiation in populations with low habitat amount. Although the method based on fine scale genetic structure may underestimate seed dispersal distance, long-distance dispersal is mainly due to pollen dispersal in many Neotropical plants (Hamilton and Miller, 2002; Ballesteros-Mejia et al., 2016). Although wind may potentially promote long-distance seed dispersal in opened areas (Tackenberg et al., 2003), landscape features may affect seed dispersal distance, seed germination and seedling recruitment (Damschen et al., 2014; Trakhtenbrot et al., 2014). In fact, sites with higher habitat amount had larger seeds (SLD, see below). Although larger seeds may increase seedling survival and thus recruitment, they may disperse to shorter distances (Ganeshaiah and Shaanker, 1991; Greene and Johnson, 1993), increasing genetic relationship within habitat fragments, and thus genetic differentiation, and decreasing genetic diversity and allelic richness.

### The Trade-Off Between Seedling Survival and Seed Dispersal May Explain the Relationships Between Adaptive Quantitative Traits and Landscape Features

Our findings show that landscape structure explained the adaptive quantitative variation of traits related to seed size and evolvability of several traits. Populations in sites with higher habitat amount tended to have larger seeds (SLD, seed longitudinal diameter). Seeds of *T. aurea* are wind dispersed and large seeds may be dispersed to short distance. There is a trade-off between seed size improving germination and seedling survival in savannas (e.g., Lahoreau et al., 2006) and dispersal (Ganeshaiah and Shaanker, 1991; Greene and Johnson, 1993). In fact, SLD is positively correlated with percentage of germination (PG, *r* = 0.767), time to germinate (TG, *r* = 0.397), plant height (IH, *r* = 0.327), and plant growth rate (HGR, *r* = 0.439). Thus, although seedlings from large seeds may growth faster, they may be dispersed to shorter distances. In the same manner, sites with higher habitat amount may provide more resources for larger seeds (Westoby et al., 1996) that may disperse to shorter distances.

On the other hand, root dry mass (RDM) and root length (RL) evolvability increased with compositional heterogeneity and RL decreased with habitat amount. In addition, RL quantitative differentiation (*Q*_*ST*_) was also higher in landscapes with higher habitat amount. In fact, root mass and length are important adaptive traits in savanna trees, which have higher root growth rates than shoot growth rates in the initial stages, allowing seedlings to obtain water from water tables (see Goldstein et al., 2008 for a review). Landscapes with higher compositional heterogeneity in agroecosystem-dominated regions may be more stressful for plant species, due to the lower habitat amount, edge effects and the presence of urban areas, roads and agriculture. In these areas, larger roots may provide higher ability to obtain water and nutrients, increasing survival, and avoid dying back because of fire (Hoffmann and Franco, 2003; Hoffmann et al., 2004). Fire is frequent in Cerrado during the dry season, and the frequency of fire is higher in agroecosystem landscapes (Pivello, 2011) that have higher spatial and temporal heterogeneity. In addition, in agroecosystems variation in soil nutrient in heterogeneous landscapes may also contribute to high variation in root size, because of selection to fast growth of roots in nutrient poor and dry areas, and relaxed selection in areas with higher nutrient inputs (for instance in edges of savanna and agriculture areas that are periodically fertilized) and moisture. The positive relationship of number of leaves (NL) additive genetic coefficient of variation and habitat amount is most likely due to the stressful environment in landscapes with lower habitat amount, similarly to RL, but in opposite direction due to negative relationship between root growth rates than shoot growth rates in the initial stages. Traits related to leaf area are correlated to plant water use efficiency, photosynthesis rate and resources retention (Westoby et al., 2002). Landscapes with lower habitat amount may be more instable leading to loss of variation in leaf traits and thus lower evolutionary potential.

## Conclusion

We found that landscape changes are affecting genetic diversity and adaptive variation in the savanna tree species *T. aurea*, despite the very recent agriculture expansion in Central Brazil (∼60 years). On one hand, the loss of habitat increases neutral genetic diversity and decreases genetic differentiation, most likely due to long distance pollen dispersal. The lower genetic differentiation between sites with lower habitat amount may also indicate that agroecosystem matrix is somehow permeable to pollinators. On the other hand, habitat amount decreases adaptive quantitative traits and increases evolvability. It is important to note that, as habitat fragmentation process (*sensu* Fahrig, 2003) is a recent event when considering the life cycle of the species, the effect of habitat loss on genetic diversity at highly variable neutral loci, such as *T. aurea*’s microsatellite loci (see Collevatti et al., 2014), may only be detected after a certain threshold of population size is attained due to Allee effect (Berec et al., 2007). As suggested for other savanna species (*C. brasiliense;* Collevatti et al., 2001), such a threshold could become dangerously small, because the high allelic variation at microsatellite loci could mask important losses of heterozygosity or very drastic events. Indeed, habitat loss had already affected some important quantitative adaptive traits, which evolve faster than neutral loci (McKay and Latta, 2002) and though may respond faster to landscape changes.

Several adaptive quantitative traits show evolutionary potential, meaning that populations still have enough variation to respond to selection, and thus to environmental changes. However, it is important to note that several populations have already low additive genetic variation for some traits at seedling stage. Evolutionary potential at short-term depends on population additive genetic variance (Hansen et al., 2011) and thus, those populations may have no evolvability for some traits, which may jeopardize species long-term persistence in these landscapes. Although mortality is higher at initial stage in savanna tree species, the low additive genetic variation for some traits may be also due to the time length of our study, because variation may be detected in other life-stages, such as juveniles. Despite the limitations of the experiment, which analyzed only seedlings and not other later stages, this result highlights the importance of the maintenance of functional connectivity among sites and landscapes, allowing the migration of advantageous alleles and thus the recovery of evolutionary potential.

## Data Availability Statement

The data and additional supporting information may be found in the online version of this article as supporting information.

## Author Contributions

RC and MR conceived and funded the work. FR and TA obtained the data. RC, JS, MR, and LC designed the experiment, field sampling, and statistical analyses. JS carried out the analyses. RC, JS, and MR wrote the original draft. All authors contributed to manuscript and approved the final version.

## Conflict of Interest

The authors declare that the research was conducted in the absence of any commercial or financial relationships that could be construed as a potential conflict of interest.
