# Supplementary material for: Multi-Scale Landscape Influences on Genetic Diversity and Adaptive Traits in a Neotropical Savanna Tree
Source: Front Genet. 2020 Mar 25;11:259. doi: 10.3389/fgene.2020.00259 (PMC7109282; doi:10.3389/fgene.2020.00259)
Supplement: Supplementary file 1 [file Table_1.DOCX]

**A multi-scale landscape analysis shows the effects of habitat amount in genetic diversity and adaptive traits in a Neotropical savanna tree**

Rosane Garcia Collevatti, Juliana Silveira dos Santos, Fernanda F Rosa, Tatiana S. Amaral, Lazaro José Chaves, Milton Cezar Ribeiro

**Appendix S1 – Tables**

**Table S1.** Landscapes and sites locality of *Tabebuia aurea* in Brazilian Cerrado. P1, P2 and P4 are structurally isolated sites in different savanna fragments, P3 and P5 are sampling sites in a single large savanna fragment in protected areas.

| **Landscape** | **Locality** | **Sites** | **Distance between sites (m)** | **Coordinates** | |
| --- | --- | --- | --- | --- | --- |
|  |  |  |  | **Latitude** | **Longitude** |
| P1 | Leopoldo de Bulhões | P1F1 | 2,383 | -16.6067 | -48.7631 |
|  |  | P1F2 |  | -16.5933 | -48.7809 |
| P2 | Vianópolis | P2F3 | 1,494 | -16.8290 | -48.5154 |
|  |  | P2F4 |  | -16.8166 | -48.5127 |
| P3 | National Forest of Silvania | P3F5 | 1,743 | -16.6358 | -48.6501 |
|  |  | P3F6 |  | -16.6414 | -48.6660 |
| P4 | Silvânia | P4F7 | 2,194 | -16.7108 | -48.7949 |
|  |  | P4F8 |  | -16.6919 | -48.7970 |
| P5 | State Park of  Caldas Novas | P5F9 | 2,601 | -17.7769 | -48.6890 |
|  |  | P5F10 |  | -17.7763 | -48.7147 |

**Table S2.** Sampling size for neutral and quantitative genetic analyses in the 10 sites of *Tabebuia aurea* in the Brazilian Cerrado. The number of adults corresponds to the number of trees sampled for neutral genetic analysis. The number of matrices corresponds to the number of trees in which fruits were collected to obtain data for adaptive quantitative genetic data.

| **Site** | **Number of adults** | **Number of matrices** | **Number of fruits** | **Number of seeds** | **Number of seedlings** |
| --- | --- | --- | --- | --- | --- |
| **P1F1** | 62 | 8 | 19 | 120 | 72 |
| **P1F2** | 57 | 5 | 9 | 75 | 46 |
| **P2F3** | 60 | 9 | 15 | 105 | 93 |
| **P2F4** | 60 | 5 | 14 | 75 | 35 |
| **P3F5** | 61 | 5 | 16 | 60 | 52 |
| **P3F6** | 61 | 4 | 7 | 45 | 26 |
| **P4F7** | 35 | 7 | 19 | 115 | 97 |
| **P4F8** | 47 | 10 | 19 | 160 | 118 |
| **P5F9** | 60 | 9 | 20 | 145 | 112 |
| **P5F10** | 60 | 10 | 20 | 170 | 115 |
| **Total** | 563 | 72 | 158 | 1070 | 766 |

**Table S3.** Adaptive quantitative traits measured in seeds and seedlings of *Tabebuia aurea* from 10 sites in the Brazilian Cerrado. The values are the average for each sampling site. SLD, seed longitudinal diameter (mm); STD, seed transversal diameter (mm); SM, seed mass (mg); TG, time to germinate; PG, proportion of seeds that germinate; IH, initial height (first measure, cm); FH, final height (last measure, cm); HGR, height growth rate (cm/day), ID, initial diameter (first measure, mm); FD, final diameter (last measure, mm); DGR, diameter growth rate (mm/day); NL, number of leaves; LL, leaf length (mm); LW, leaf width (mm); RL, root length (cm); RGM, root green mass (g), RDM, root dry mass (g); ASL, aboveground shoot length (plant height, cm); ADM, aboveground dry mass (g); AGM, aboveground green mass (g).

| **Site** | **SLD** | **STD** | **SM** | **TG** | **PG** | **IH** | **FH** | **HGR** | **ID** | **FD** | **DGR** | **NL** | **LL** | **LW** | **RL** | **RGM** | **RDM** | **ASL** | **ADM** | **AGM** |
| --- | --- | --- | --- | --- | --- | --- | --- | --- | --- | --- | --- | --- | --- | --- | --- | --- | --- | --- | --- | --- |
| P1F1 | 54.67 | 17.91 | 0.20 | 11.15 | 0.66 | 1.28 | 2.25 | 0.41 | 3.21 | 3.09 | -0.05 | 4.79 | 81.52 | 23.88 | 21.80 | 6.08 | 1.49 | 11.47 | 1.41 | 0.51 |
| P1F2 | 57.04 | 16.95 | 0.18 | 11.00 | 0.63 | 1.15 | 2.29 | 0.49 | 2.84 | 2.63 | -0.10 | 5.87 | 68.05 | 20.51 | 22.87 | 3.45 | 0.99 | 9.84 | 1.12 | 0.45 |
| P2F3 | 57.28 | 18.00 | 0.20 | 11.50 | 1.00 | 1.28 | 2.28 | 0.43 | 3.00 | 2.85 | -0.10 | 4.72 | 82.62 | 22.41 | 22.50 | 4.67 | 1.37 | 11.62 | 1.18 | 0.49 |
| P2F4 | 54.76 | 15.69 | 0.26 | 11.00 | 0.71 | 1.69 | 2.51 | 0.35 | 2.95 | 2.82 | -0.16 | 4.37 | 76.10 | 23.25 | 20.57 | 5.20 | 1.46 | 11.27 | 1.96 | 0.46 |
| P3F5 | 54.26 | 16.78 | 0.18 | 10.29 | 0.98 | 1.21 | 2.21 | 0.44 | 2.82 | 2.93 | -0.01 | 4.64 | 69.27 | 23.93 | 20.57 | 4.55 | 1.22 | 10.31 | 1.18 | 0.45 |
| P3F6 | 58.67 | 18.13 | 0.25 | 12.67 | 0.84 | 1.42 | 2.22 | 0.33 | 3.12 | 2.76 | -0.16 | 4.56 | 64.63 | 21.28 | 19.73 | 3.87 | 1.32 | 9.56 | 0.94 | 0.39 |
| P4F7 | 50.33 | 16.61 | 0.26 | 11.86 | 0.95 | 1.06 | 2.27 | 0.52 | 3.28 | 3.27 | -0.01 | 5.08 | 91.90 | 25.29 | 24.54 | 7.15 | 1.82 | 12.77 | 0.77 | 2.13 |
| P4F8 | 53.02 | 17.06 | 0.22 | 11.72 | 0.96 | 1.04 | 2.32 | 0.55 | 3.18 | 3.23 | 0.02 | 5.19 | 83.56 | 25.89 | 24.11 | 7.13 | 1.88 | 11.89 | 0.70 | 1.97 |
| P5F9 | 56.99 | 17.03 | 0.22 | 11.11 | 0.85 | 1.22 | 2.26 | 0.45 | 3.26 | 3.19 | -0.04 | 5.20 | 82.09 | 23.46 | 24.76 | 6.79 | 1.78 | 11.73 | 0.69 | 1.94 |
| P5F10 | 58.54 | 17.79 | 0.22 | 11.73 | 0.92 | 1.17 | 2.33 | 0.50 | 3.18 | 3.14 | -0.01 | 4.51 | 86.35 | 26.07 | 23.29 | 7.57 | 2.06 | 12.15 | 0.69 | 1.83 |

**Table S4** Correlation among adaptive quantitative traits measured in seeds and seedlings of *Tabebuia aurea* from 10 sites (node level analysis) in the Brazilian Cerrado. The values are the average for each sampling site. SLD, seed longitudinal diameter (mm); STD, seed transversal diameter (mm); SM, seed mass (mg); TG, time to germinate; PG, proportion of seeds that germinate; IH, initial height (first measure, cm); FH, final height (last measure, cm); HGR, height growth rate (cm/day), ID, initial diameter (first measure, mm); FD, final diameter (last measure, mm); DGR, diameter growth rate (mm/day); NL, number of leaves; LL, leaf length (mm); LW, leaf width (mm); RL, root length (cm); RGM, root green mass (g), RDM, root dry mass (g); ASL, aboveground shoot length (plant height, cm); ADM, aboveground dry mass (g); AGM, aboveground green mass (g).

|  | **SLD** | **STD** | **SM** | **TG** | **PG** | **IH** | **FH** | **HGR** | **ID** | **FD** | **DGR** | **NL** | **LL** | **LW** | **RL** | **RGM** | **RDM** | **ASL** | **ADM** | **AGM** |
| --- | --- | --- | --- | --- | --- | --- | --- | --- | --- | --- | --- | --- | --- | --- | --- | --- | --- | --- | --- | --- |
| **SLD** | 1.000 | 0.046 | -0.205 | 0.397 | 0.767 | 0.323 | 0.215 | 0.439 | -0.163 | -0.689 | -0.086 | 0.106 | -0.277 | 0.189 | -0.315 | -0.432 | -0.160 | -0.167 | -0.064 | -0.338 |
| **STD** | 0.046 | 1.000 | -0.076 | 0.122 | -0.254 | 0.436 | 0.366 | 0.349 | -0.306 | 0.277 | 0.126 | -0.318 | 0.176 | -0.026 | -0.595 | -0.147 | -0.216 | 0.392 | 0.020 | -0.281 |
| **SM** | -0.205 | -0.076 | 1.000 | -0.194 | -0.243 | -0.242 | 0.005 | 0.066 | -0.237 | -0.014 | 0.723 | 0.156 | 0.404 | -0.219 | 0.477 | 0.527 | 0.453 | 0.083 | 0.771 | 0.189 |
| **TG** | 0.397 | 0.122 | -0.194 | 1.000 | 0.562 | 0.503 | -0.318 | 0.005 | -0.059 | -0.334 | -0.012 | -0.327 | -0.111 | 0.475 | -0.460 | -0.358 | -0.042 | -0.270 | -0.021 | -0.533 |
| **PG** | 0.767 | -0.254 | -0.243 | 0.562 | 1.000 | 0.444 | 0.023 | 0.401 | -0.117 | -0.869 | -0.034 | 0.395 | -0.472 | 0.283 | -0.021 | -0.451 | -0.334 | -0.364 | -0.010 | -0.420 |
| **IH** | 0.323 | 0.436 | -0.242 | 0.503 | 0.444 | 1.000 | 0.510 | 0.753 | 0.040 | -0.354 | 0.326 | 0.000 | -0.613 | 0.348 | -0.587 | -0.428 | -0.316 | -0.269 | 0.259 | -0.866 |
| **FH** | 0.215 | 0.366 | 0.005 | -0.318 | 0.023 | 0.510 | 1.000 | 0.809 | 0.279 | 0.014 | 0.503 | 0.408 | -0.273 | -0.108 | -0.305 | 0.198 | 0.007 | 0.261 | 0.370 | -0.219 |
| **HGR** | 0.439 | 0.349 | 0.066 | 0.005 | 0.401 | 0.753 | 0.809 | 1.000 | -0.064 | -0.457 | 0.547 | 0.419 | -0.563 | -0.062 | -0.214 | -0.166 | -0.209 | -0.149 | 0.490 | -0.596 |
| **ID** | -0.163 | -0.306 | -0.237 | -0.059 | -0.117 | 0.040 | 0.279 | -0.064 | 1.000 | 0.435 | -0.011 | 0.074 | -0.022 | 0.394 | -0.287 | 0.378 | 0.173 | 0.239 | -0.179 | 0.212 |
| **FD** | -0.689 | 0.277 | -0.014 | -0.334 | -0.869 | -0.354 | 0.014 | -0.457 | 0.435 | 1.000 | -0.068 | -0.345 | 0.567 | -0.086 | -0.206 | 0.547 | 0.309 | 0.591 | -0.201 | 0.504 |
| **DGR** | -0.086 | 0.126 | 0.723 | -0.012 | -0.034 | 0.326 | 0.503 | 0.547 | -0.011 | -0.068 | 1.000 | 0.343 | 0.013 | -0.263 | 0.153 | 0.534 | 0.490 | 0.014 | 0.961 | -0.218 |
| **NL** | 0.106 | -0.318 | 0.156 | -0.327 | 0.395 | 0.000 | 0.408 | 0.419 | 0.074 | -0.345 | 0.343 | 1.000 | -0.122 | -0.291 | 0.573 | 0.350 | -0.120 | 0.173 | 0.274 | 0.252 |
| **LL** | -0.277 | 0.176 | 0.404 | -0.111 | -0.472 | -0.613 | -0.273 | -0.563 | -0.022 | 0.567 | 0.013 | -0.122 | 1.000 | -0.043 | 0.117 | 0.592 | 0.302 | 0.769 | -0.070 | 0.749 |
| **LW** | 0.189 | -0.026 | -0.219 | 0.475 | 0.283 | 0.348 | -0.108 | -0.062 | 0.394 | -0.086 | -0.263 | -0.291 | -0.043 | 1.000 | -0.514 | -0.386 | -0.448 | 0.062 | -0.335 | -0.218 |
| **RL** | -0.315 | -0.595 | 0.477 | -0.460 | -0.021 | -0.587 | -0.305 | -0.214 | -0.287 | -0.206 | 0.153 | 0.573 | 0.117 | -0.514 | 1.000 | 0.346 | 0.130 | -0.131 | 0.264 | 0.467 |
| **RGM** | -0.432 | -0.147 | 0.527 | -0.358 | -0.451 | -0.428 | 0.198 | -0.166 | 0.378 | 0.547 | 0.534 | 0.350 | 0.592 | -0.386 | 0.346 | 1.000 | 0.682 | 0.537 | 0.420 | 0.633 |
| **RDM** | -0.160 | -0.216 | 0.453 | -0.042 | -0.334 | -0.316 | 0.007 | -0.209 | 0.173 | 0.309 | 0.490 | -0.120 | 0.302 | -0.448 | 0.130 | 0.682 | 1.000 | -0.021 | 0.525 | 0.197 |
| **ASL** | -0.167 | 0.392 | 0.083 | -0.270 | -0.364 | -0.269 | 0.261 | -0.149 | 0.239 | 0.591 | 0.014 | 0.173 | 0.769 | 0.062 | -0.131 | 0.537 | -0.021 | 1.000 | -0.191 | 0.661 |
| **ADM** | -0.064 | 0.020 | 0.771 | -0.021 | -0.010 | 0.259 | 0.370 | 0.490 | -0.179 | -0.201 | 0.961 | 0.274 | -0.070 | -0.335 | 0.264 | 0.420 | 0.525 | -0.191 | 1.000 | -0.285 |
| **AGM** | -0.338 | -0.281 | 0.189 | -0.533 | -0.420 | -0.866 | -0.219 | -0.596 | 0.212 | 0.504 | -0.218 | 0.252 | 0.749 | -0.218 | 0.467 | 0.633 | 0.197 | 0.661 | -0.285 | 1.000 |

**Table S5** Correlation among adaptive quantitative traits measured in seeds and seedlings of *Tabebuia aurea* from 5 landscapes (link level analysis) in the Brazilian Cerrado. The values are the average for each sampling site. SLD, seed longitudinal diameter (mm); STD, seed transversal diameter (mm); SM, seed mass (mg); TG, time to germinate; PG, proportion of seeds that germinate; IH, initial height (first measure, cm); FH, final height (last measure, cm); HGR, height growth rate (cm/day), ID, initial diameter (first measure, mm); FD, final diameter (last measure, mm); DGR, diameter growth rate (mm/day); NL, number of leaves; LL, leaf length (mm); LW, leaf width (mm); RL, root length (cm); RGM, root green mass (g), RDM, root dry mass (g); ASL, aboveground shoot length (plant height, cm); ADM, aboveground dry mass (g); AGM, aboveground green mass (g).

|  | **SLD** | **STD** | **SM** | **TG** | **PG** | **IH** | **FH** | **HGR** | **ID** | **FD** | **DGR** | **NL** | **LL** | **LW** | **RL** | **RGM** | **RDM** | **ASL** | **ADM** | **AGM** |
| --- | --- | --- | --- | --- | --- | --- | --- | --- | --- | --- | --- | --- | --- | --- | --- | --- | --- | --- | --- | --- |
| **SLD** | 1.000 | 0.073 | -0.496 | 0.719 | 0.952 | 0.516 | 0.407 | 0.716 | -0.091 | -0.888 | 0.115 | 0.715 | -0.113 | 0.455 | -0.239 | -0.253 | -0.530 | 0.082 | 0.068 | -0.391 |
| **STD** | 0.073 | 1.000 | 0.550 | 0.138 | -0.103 | 0.581 | 0.821 | 0.653 | -0.957 | 0.085 | 0.992 | -0.365 | 0.145 | -0.404 | -0.680 | -0.101 | -0.016 | 0.298 | 0.998 | -0.398 |
| **SM** | -0.496 | 0.550 | 1.000 | -0.485 | -0.658 | -0.230 | 0.077 | -0.116 | -0.636 | 0.704 | 0.585 | -0.382 | 0.613 | -0.831 | 0.114 | 0.449 | 0.370 | 0.466 | 0.517 | 0.325 |
| **TG** | 0.719 | 0.138 | -0.485 | 1.000 | 0.843 | 0.846 | 0.226 | 0.809 | 0.044 | -0.915 | 0.098 | 0.101 | -0.686 | 0.790 | -0.632 | -0.836 | -0.946 | -0.565 | 0.164 | -0.871 |
| **PG** | 0.952 | -0.103 | -0.658 | 0.843 | 1.000 | 0.560 | 0.214 | 0.667 | 0.150 | -0.980 | -0.088 | 0.611 | -0.372 | 0.700 | -0.273 | -0.470 | -0.709 | -0.215 | -0.095 | -0.520 |
| **IH** | 0.516 | 0.581 | -0.230 | 0.846 | 0.560 | 1.000 | 0.615 | 0.925 | -0.375 | -0.662 | 0.521 | -0.227 | -0.622 | 0.508 | -0.939 | -0.808 | -0.724 | -0.416 | 0.614 | -0.959 |
| **FH** | 0.407 | 0.821 | 0.077 | 0.226 | 0.214 | 0.615 | 1.000 | 0.717 | -0.813 | -0.206 | 0.824 | 0.012 | 0.087 | -0.192 | -0.674 | -0.076 | 0.047 | 0.386 | 0.826 | -0.393 |
| **HGR** | 0.716 | 0.653 | -0.116 | 0.809 | 0.667 | 0.925 | 0.717 | 1.000 | -0.538 | -0.697 | 0.639 | 0.063 | -0.319 | 0.328 | -0.793 | -0.569 | -0.646 | -0.101 | 0.665 | -0.796 |
| **ID** | -0.091 | -0.957 | -0.636 | 0.044 | 0.150 | -0.375 | -0.813 | -0.538 | 1.000 | -0.188 | -0.983 | 0.170 | -0.418 | 0.600 | 0.459 | -0.177 | -0.185 | -0.560 | -0.940 | 0.144 |
| **FD** | -0.888 | 0.085 | 0.704 | -0.915 | -0.980 | -0.662 | -0.206 | -0.697 | -0.188 | 1.000 | 0.096 | -0.454 | 0.545 | -0.805 | 0.403 | 0.631 | 0.796 | 0.384 | 0.067 | 0.658 |
| **DGR** | 0.115 | 0.992 | 0.585 | 0.098 | -0.088 | 0.521 | 0.824 | 0.639 | -0.983 | 0.096 | 1.000 | -0.267 | 0.253 | -0.469 | -0.599 | -0.003 | 0.032 | 0.402 | 0.983 | -0.314 |
| **NL** | 0.715 | -0.365 | -0.382 | 0.101 | 0.611 | -0.227 | 0.012 | 0.063 | 0.170 | -0.454 | -0.267 | 1.000 | 0.396 | 0.077 | 0.477 | 0.394 | 0.033 | 0.469 | -0.396 | 0.347 |
| **LL** | -0.113 | 0.145 | 0.613 | -0.686 | -0.372 | -0.622 | 0.087 | -0.319 | -0.418 | 0.545 | 0.253 | 0.396 | 1.000 | -0.878 | 0.609 | 0.945 | 0.718 | 0.940 | 0.091 | 0.807 |
| **LW** | 0.455 | -0.404 | -0.831 | 0.790 | 0.700 | 0.508 | -0.192 | 0.328 | 0.600 | -0.805 | -0.469 | 0.077 | -0.878 | 1.000 | -0.351 | -0.820 | -0.792 | -0.815 | -0.363 | -0.667 |
| **RL** | -0.239 | -0.680 | 0.114 | -0.632 | -0.273 | -0.939 | -0.674 | -0.793 | 0.459 | 0.403 | -0.599 | 0.477 | 0.609 | -0.351 | 1.000 | 0.752 | 0.509 | 0.396 | -0.719 | 0.907 |
| **RGM** | -0.253 | -0.101 | 0.449 | -0.836 | -0.470 | -0.808 | -0.076 | -0.569 | -0.177 | 0.631 | -0.003 | 0.394 | 0.945 | -0.820 | 0.752 | 1.000 | 0.858 | 0.871 | -0.149 | 0.940 |
| **RDM** | -0.530 | -0.016 | 0.370 | -0.946 | -0.709 | -0.724 | 0.047 | -0.646 | -0.185 | 0.796 | 0.032 | 0.033 | 0.718 | -0.792 | 0.509 | 0.858 | 1.000 | 0.705 | -0.040 | 0.814 |
| **ASL** | 0.082 | 0.298 | 0.466 | -0.565 | -0.215 | -0.416 | 0.386 | -0.101 | -0.560 | 0.384 | 0.402 | 0.469 | 0.940 | -0.815 | 0.396 | 0.871 | 0.705 | 1.000 | 0.251 | 0.656 |
| **ADM** | 0.068 | 0.998 | 0.517 | 0.164 | -0.095 | 0.614 | 0.826 | 0.665 | -0.940 | 0.067 | 0.983 | -0.396 | 0.091 | -0.363 | -0.719 | -0.149 | -0.040 | 0.251 | 1.000 | -0.440 |
| **AGM** | -0.391 | -0.398 | 0.325 | -0.871 | -0.520 | -0.959 | -0.393 | -0.796 | 0.144 | 0.658 | -0.314 | 0.347 | 0.807 | -0.667 | 0.907 | 0.940 | 0.814 | 0.656 | -0.440 | 1.000 |

**Table S6** Landscape metrics measured in buffers of 0.5 km in 10 sites of *Tabebuia aurea* from 5 landscapes (node level analysis) in the Brazilian Cerrado.

| **Node** | **Habitat amount** | **Composition heterogeneity** | **BMQ** | **Functional connectivity(ha)** |
| --- | --- | --- | --- | --- |
| **P1F1** | 36.31 | 1.50 | 0.57 | 781.13 |
| **P1F2** | 13.02 | 1.31 | 0.45 | 169.30 |
| **P2F3** | 31.97 | 0.94 | 0.54 | 191.65 |
| **P2F4** | 16.22 | 0.49 | 0.42 | 540.88 |
| **P3F5** | 39.18 | 1.57 | 0.64 | 986.20 |
| **P3F6** | 39.18 | 1.57 | 0.64 | 986.20 |
| **P4F7** | 17.81 | 1.45 | 0.45 | 315.25 |
| **P4F8** | 14.31 | 1.23 | 0.45 | 154.03 |
| **P5F9** | 76.70 | 0.85 | 0.84 | 18206.00 |
| **P5F10** | 76.70 | 0.85 | 0.84 | 18206.00 |

**Table S7** Landscape metrics measured in buffers of 2.0, 4.0 and 6 km in 5 landscapes (link level analysis) of *Tabebuia aurea* in the Brazilian Cerrado. HA-2, habitat amount at 2 km buffer; BMQ-2, Buffer Matrix Quality at 2 km buffer; CH-2, composition heterogeneity at 2 km buffer; FC-2, functional connectivity at 2 km buffer; SA-2, savannah amount at 2 km buffer; SA+PA+WE-2, amount of savanna+pasture+wetlands at 2 km buffer; SA+PA+WE+SE+RI-2, amount of savanna+pasture+wetlands+seasonal forests+riparian forests at 2 km buffer; HA-4, habitat amount at 4 km buffer; BMQ-4, Buffer Matrix Quality at 4 km buffer; CH-4, composition heterogeneity at 4 km buffer; FC-4, functional connectivity at 4 km buffer; SA-4, savanna amount at 4 km buffer; SA+PA+WE-4, amount of savanna+pasture+wetlands at 4 km buffer; SA+PA+WE+SE+RI-4, amount of savanna+pasture+wetlands+seasonal forests+riparian forests at 4 km buffer; SA4-2-4, savanna amount at 4 minus 2km buffers; SA+PA+WE4-2-4, amount of savanna+pasture+wetlands at 4 minus 2km buffers; SA+PA+WE+SE+RI4-2-4, amount of savanna+pasture+wetlands+seasonal forests+riparian forests at 4 minus 2km buffers; HA-6, habitat amount at 6 km buffer; BMQ-6, Buffer Matrix Quality at 6 km buffer; CH-6, composition heterogeneity at 6 km buffer; FC-6, functional connectivity at 6 km buffer; SA-6, savanna amount at 6 km buffer; SA+PA+WE-6, amount of savanna+pasture+wetlands at 6 km buffer; SA+PA+WE+SE+RI-6, amount of savanna+pasture+wetlands+seasonal forests+riparian forests at 6 km buffer; SA6-4-6, savanna amount at 6 minus 4km buffers; SA+PA+WE6-4-6, amount of savanna+pasture+wetlands at 6 minus 4km buffers; SA+PA+WE+SE+RI6-4-6, amount of savanna+pasture+wetlands+seasonal forests+riparian forests at 6 minus 4km buffers;

| **Landscape** | HA-2 | BMQ-2 | CA-2 | FC-2 | SA-2 | SA+PA+WE_2 | SA+PA+WE+SE+RI _2 | HAt_4 | BMQ_4 | CH_4 | FC_4 | SA_4 | SA+PA+WE_4 | SA+PA+WE+SE+RI_4 | SA4_2_4 | SA+PA+WE4_2_4 | SA+PA+WE+SE+RI 4_2_4 |
| --- | --- | --- | --- | --- | --- | --- | --- | --- | --- | --- | --- | --- | --- | --- | --- | --- | --- |
| **P1** | 28.96 | 0.53 | 1.47 | 408140.00 | 363.87 | 576.48 | 760.57 | 11.16 | 0.41 | 1.61 | 902784.00 | 560.82 | 1719.78 | 2566.62 | 196.95 | 1143.31 | 1806.06 |
| **P2** | 23.30 | 0.48 | 0.89 | 333922.00 | 292.79 | 320.62 | 363.91 | 8.45 | 0.38 | 1.06 | 769658.00 | 424.72 | 887.78 | 1439.58 | 131.93 | 567.15 | 1075.68 |
| **P3** | 33.84 | 0.59 | 1.62 | 431704.00 | 425.16 | 651.90 | 958.01 | 15.02 | 0.44 | 1.55 | 970983.00 | 755.07 | 1760.24 | 2661.63 | 329.91 | 1108.34 | 1703.62 |
| **P4** | 15.40 | 0.43 | 1.45 | 378859.00 | 193.53 | 460.97 | 606.41 | 8.89 | 0.37 | 1.39 | 982315.00 | 446.61 | 1996.21 | 2699.02 | 253.09 | 1535.24 | 2092.61 |
| **P5** | 100.00 | 1.00 | 0.00 | 600321.00 | 1256.55 | 1256.55 | 1256.55 | 100.00 | 1.00 | 0.00 | 2245905.00 | 5026.49 | 5026.49 | 5026.49 | 3769.94 | 3769.94 | 3769.94 |

**Table S7 Continuing**

| **Landscape** | HA_6 | BMQ_6 | CH_6 | FC_6 | SA_6 | SA+PA+WE_6 | SA+PA+WE+SE+RI_6 | SA6_4_6 | SA+PA+WE6_4_6 | SA+PA+WE+SE+RI6_4_6 |
| --- | --- | --- | --- | --- | --- | --- | --- | --- | --- | --- |
| **P1** | 8.47 | 0.39 | 1.58 | 1605608.00 | 958.13 | 4216.42 | 6465.27 | 397.32 | 2496.64 | 3898.65 |
| **P2** | 6.04 | 0.37 | 1.10 | 1260531.00 | 682.53 | 1780.04 | 3179.78 | 257.81 | 892.26 | 1740.20 |
| **P3** | 10.51 | 0.41 | 1.61 | 2066757.00 | 1188.54 | 4088.96 | 6197.85 | 433.47 | 2328.72 | 3536.22 |
| **P4** | 5.40 | 0.34 | 1.36 | 1477864.00 | 611.43 | 4565.10 | 6241.38 | 164.81 | 2568.89 | 3542.36 |
| **P5** | 89.13 | 0.93 | 0.48 | 4677359.00 | 10079.94 | 10705.09 | 10999.22 | 5053.44 | 5678.60 | 5972.73 |

**Table S8** Correlation among landscape metrics measured in buffers of 2.0 km in 5 landscapes (link level analysis) of *Tabebuia aurea* in the Brazilian Cerrado. BMQ, Buffer Matrix Quality; Savanna, is the amount of savanna at 2 km buffer; SA+PA+WE, is the amount of savanna+pasture+wetlands at 2 km buffer; SA+PA+WE+SE+RI, is the amount of savanna+pasture+wetlands+seasonal forests+riparian forests at 2 km buffer

|  | **Habitat amount** | **Composition heterogeneity** | **BMQ** | **Functional connectivity** | **Savanna** | **SA+PA+WE** | **SA+PA+WE+SE+RI** |
| --- | --- | --- | --- | --- | --- | --- | --- |
| **Habitat amount** | 1.000 | -0.862 | 0.997 | 0.961 | 1.000 | 0.966 | 0.840 |
| **Composition heterogeneity** | -0.862 | 1.000 | -0.826 | -0.708 | -0.862 | -0.718 | -0.453 |
| **BMQ** | 0.997 | -0.826 | 1.000 | 0.970 | 0.997 | 0.974 | 0.871 |
| **Functional connectivity** | 0.961 | -0.708 | 0.970 | 1.000 | 0.961 | 1.000 | 0.946 |
| **Savanna** | 1.000 | -0.862 | 0.997 | 0.961 | 1.000 | 0.966 | 0.840 |
| **SA+PA+WE** | 0.966 | -0.718 | 0.974 | 1.000 | 0.966 | 1.000 | 0.942 |
| **SA+PA+WE+SE+RI** | 0.840 | -0.453 | 0.871 | 0.946 | 0.840 | 0.942 | 1.000 |

**Table S9** Correlation among landscape metrics measured in buffers of 0.5 km in 10 sites in 5 landscapes (node level analysis) of *Tabebuia aurea* in the Brazilian Cerrado. BMQ, Buffer Matrix Quality.

|  | **Habitat amount** | **Composition heterogeneity** | **BMQ** | **Functional connectivity** |
| --- | --- | --- | --- | --- |
| **Habitat amount** | 1 | -0.236439473 | 0.988999293 | 0.914484274 |
| **Composition heterogeneity** | -0.236439473 | 1 | -0.13138389 | -0.446917008 |
| **BMQ** | 0.988999293 | -0.13138389 | 1 | 0.879570163 |
| **Functional connectivity** | 0.914484274 | -0.446917008 | 0.879570163 | 1 |

**Table S10.** Neutral genetic variability for 10 sites in 5 landscapes of *Tabebuia aurea* in the Brazilian Cerrado. He, genetic diversity; AR, allelic richness; f, inbreeding coefficient; Ne, effective population size. *f* values are significant for all sites (p < 0.05).

| **Site** | ***He*** | ***AR*** | ***f*** | ***Ne*** |
| --- | --- | --- | --- | --- |
| P1F1 | 0.921 | 17.760 | 0.121 | 53.600 |
| P1F2 | 0.904 | 16.000 | 0.138 | 19.900 |
| P2F3 | 0.920 | 17.170 | 0.156 | 19.300 |
| P2F4 | 0.919 | 18.453 | 0.203 | 49.400 |
| P3F5 | 0.885 | 14.949 | 0.207 | 51.500 |
| P3F6 | 0.912 | 18.220 | 0.180 | 56.000 |
| P4F7 | 0.915 | 17.943 | 0.231 | 19.700 |
| P4F8 | 0.898 | 15.965 | 0.151 | 32.600 |
| P5F9 | 0.874 | 14.188 | 0.141 | 52.900 |
| P5F10 | 0.869 | 14.373 | 0.171 | 51.900 |

**Table S11.** Neutral genetic differentiation and inbreeding coefficient (*F_IS_*) for 5 landscapes of *Tabebuia aurea* in the Brazilian Cerrado. All values are significant for all sites (p < 0.05).

| ***Landscape*** | ***F_ST_*** | ***G_ST_*** | **Jost's D** | ***F_IS_*** |
| --- | --- | --- | --- | --- |
| **P1** | 0.011 | 0.155 | 0.142 | 0.130 |
| **P2** | 0.012 | 0.156 | 0.144 | 0.180 |
| **P3** | 0.020 | 0.304 | 0.277 | 0.194 |
| **P4** | 0.008 | 0.115 | 0.104 | 0.191 |
| **P5** | 0.024 | 0.281 | 0.248 | 0.156 |

**Table S12.** Additive genetic coefficient of variation (*CV_a_%*), narrow heritability (*h*), additive genetic variance (*V_a_*) and residual variance (R_V_) of adaptive quantitative traits measured in seedlings of *Tabebuia aurea* from 10 sites in the Brazilian Cerrado. NL, number of leaves; LW, leaf width (mm); RL, root length (cm); RDM, root dry mass (g); ASL, aboveground shoot length (plant height, cm).

| **Site** | **NL** | | | | **LW** | | | | **RL** | | | | **RDM** | | | | **ASL** | | | |
| --- | --- | --- | --- | --- | --- | --- | --- | --- | --- | --- | --- | --- | --- | --- | --- | --- | --- | --- | --- | --- |
|  | ***CV_a_%*** | ***h^2^*** | ***V_a_*** | ***R_V_*** | ***CV_a_%*** | ***h^2^*** | ***V_a_*** | ***R_V_*** | ***CV_a_%*** | ***h^2^*** | ***V_a_*** | ***R_V_*** | ***CV_a_%*** | ***h^2^*** | ***V_a_*** | ***R_V_*** | ***CV_a_%*** | ***h^2^*** | ***V_a_*** | ***R_V_*** |
| **P1F1** | 28.77 | 0.4322 | 1.9754 | 3.752 | 3.86 | 0.0246 | 0.8485 | 35.183 | 5.83 | 0.0922 | 1.6129 | 16.833 | 19.96 | 0.1989 | 0.0884 | 0.4076 | 0.98 | 0.0021 | 0.0127 | 6.0679 |
| **P1F2** | 2.80 | 0.0068 | 0.0283 | 4.173 | 24.38 | 0.9419 | 23.4227 | 14.580 | 13.21 | 0.4268 | 9.3969 | 17.890 | 36.07 | 0.3249 | 0.1219 | 0.3196 | 14.97 | 0.4545 | 2.0849 | 3.6713 |
| **P2F3** | 18.93 | 0.2682 | 0.7631 | 2.490 | 14.94 | 0.4026 | 10.6164 | 21.422 | 4.62 | 0.0852 | 1.0828 | 12.204 | 29.00 | 0.3248 | 0.1499 | 0.3917 | 12.14 | 0.2578 | 1.9753 | 6.7417 |
| **P2F4** | 2.76 | 0.0031 | 0.0145 | 4.706 | 0.94 | 0.0023 | 0.0479 | 20.441 | 1.25 | 0.0060 | 0.0695 | 11.540 | 3.450 | 0.0024 | 0.0025 | 1.0616 | 5.95 | 0.0857 | 0.4561 | 5.0775 |
| **P3F5** | 12.30 | 0.1136 | 0.3447 | 2.846 | 21.66 | 0.7349 | 25.9293 | 21.177 | 7.70 | 0.1322 | 2.4891 | 17.474 | 49.70 | 0.7137 | 0.3331 | 0.2854 | 16.40 | 0.5310 | 2.7771 | 3.7183 |
| **P3F6** | 18.94 | 0.4821 | 0.7713 | 1.212 | 1.63 | 0.0045 | 0.1204 | 27.003 | 5.61 | 0.0668 | 1.2173 | 17.617 | 47.65 | 0.4053 | 0.3659 | 0.7190 | 20.29 | 0.8437 | 3.8953 | 2.6600 |
| **P4F7** | 14.72 | 0.1550 | 0.5799 | 3.403 | 2.96 | 0.0185 | 0.5587 | 29.942 | 3.85 | 0.0376 | 0.8918 | 23.191 | 20.23 | 0.2672 | 0.1350 | 0.4267 | 11.42 | 0.2752 | 2.1236 | 6.4791 |
| **P4F8** | 17.44 | 0.2016 | 0.8203 | 3.693 | 5.42 | 0.1090 | 1.9731 | 17.201 | 10.56 | 0.2865 | 6.4794 | 19.646 | 42.03 | 0.6877 | 0.6116 | 0.6088 | 9.02 | 0.1862 | 1.1547 | 5.6711 |
| **P5F9** | 27.91 | 0.4313 | 2.1599 | 4.050 | 6.80 | 0.1326 | 2.5404 | 18.022 | 0.96 | 0.0023 | 0.0570 | 25.221 | 35.40 | 0.5843 | 0.3884 | 0.4986 | 20.04 | 0.7813 | 5.5307 | 4.6253 |
| **P5F10** | 16.90 | 0.1570 | 0.5878 | 3.458 | 8.21 | 0.1567 | 4.5025 | 26.540 | 0.97 | 0.0034 | 0.0510 | 15.114 | 21.88 | 0.2040 | 0.2074 | 0.9149 | 8.07 | 0.1222 | 0.9571 | 7.3660 |

**Table S13.** Additive genetic differentiation of adaptive quantitative traits measured in seeds (*P_ST_*) and seedlings (*Q_ST_*) of *Tabebuia aurea* from 5 landscapes in the Brazilian Cerrado. SLD, seed longitudinal diameter; STD, seed transversal diameter; SM, seed mass; NL, number of leaves; LW, leaf width; RL, root length; RDM, root dry mass; ASL, aboveground shoot length (plant height).

| **Landscape** | ***P_ST_*** | | | ***Q_ST_*** | | | | |
| --- | --- | --- | --- | --- | --- | --- | --- | --- |
|  | **SLD** | **STD** | **SM** | **NL** | **LW** | **RL** | **RDM** | **ASL** |
| **P1** | 0.0342 | 0.0006 | 0.0055 | 0.1196 | 0.1562 | 0.0201 | 0.2868 | 0.4457 |
| **P2** | 0.0003 | 0.0006 | 0.0835 | 0.0052 | 0.0016 | 0.1139 | 0.0024 | 0.0013 |
| **P3** | 0.0390 | 0.0615 | 0.0602 | 0.0022 | 0.0010 | 0.0042 | 0.0008 | 0.0009 |
| **P4** | 0.0026 | 0.0002 | 0.0346 | 0.0008 | 0.0055 | 0.0013 | 0.0005 | 0.0356 |
| **P5** | 0.0003 | 0.0078 | 0.0005 | 0.0362 | 0.1751 | 0.8140 | 0.0300 | 0.0006 |
